# An insight into the sialotranscriptome of the brown dog tick, *Rhipicephalus sanguineus*

**DOI:** 10.1186/1471-2164-11-450

**Published:** 2010-07-22

**Authors:** Elen Anatriello, José MC Ribeiro, Isabel KF de Miranda-Santos, Lucinda G Brandão, Jennifer M Anderson, Jesus G Valenzuela, Sandra R Maruyama, João S Silva, Beatriz R Ferreira

**Affiliations:** 1 Department of Biochemistry and Immunology, Ribeirão Preto School of Medicine, University of São Paulo, Ribeirão Preto, SP 14049-900, Brazil; 2 Department of Maternal and Child and Public Health Nursing, Ribeirão Preto School of Nursing, University of São Paulo, Ribeirão Preto, SP 14049-900, Brazil; 3 Laboratory of Malaria and Vector Research, National Institute of Allergy and Infectious Diseases, National Institutes of Health, Bethesda, MD 20892-8132, USA; 4 Embrapa Recursos Genéticos e Biotecnologia, 70770-900, Brasília, DF, Brazil; 5 Universidade Paulista, Avenida Baguaçu, 1939, 16018-280, Araçatuba, SP, Brasil

## Abstract

**Background:**

*Rhipicephalus sanguineus*, known as the brown dog tick, is a common ectoparasite of domestic dogs and can be found worldwide. *R.sanguineus *is recognized as the primary vector of the etiological agent of canine monocytic ehrlichiosis and canine babesiosis. Here we present the first description of a *R. sanguineus *salivary gland transcriptome by the production and analysis of 2,034 expressed sequence tags (EST) from two cDNA libraries, one consctructed using mRNA from dissected salivary glands from female ticks fed for 3-5 days (early to mid library, RsSGL1) and the another from ticks fed for 5 days (mid library, RsSGL2), identifying 1,024 clusters of related sequences.

**Results:**

Based on sequence similarities to nine different databases, we identified transcripts of genes that were further categorized according to function. The category of putative housekeeping genes contained ~56% of the sequences and had on average 2.49 ESTs per cluster, the secreted protein category contained 26.6% of the ESTs and had 2.47 EST's/clusters, while 15.3% of the ESTs, mostly singletons, were not classifiable, and were annotated as "unknown function". The secreted category included genes that coded for lipocalins, proteases inhibitors, disintegrins, metalloproteases, immunomodulatory and antiinflammatory proteins, as Evasins and Da-p36, as well as basic-tail and 18.3 kDa proteins, cement proteins, mucins, defensins and antimicrobial peptides. Comparison of the abundance of ESTs from similar contigs of the two salivary gland cDNA libraries allowed the identification of differentially expressed genes, such as genes coding for Evasins and a thrombin inhibitor, which were over expressed in the RsSGL1 (early to mid library) versus RsSGL2 (mid library), indicating their role in inhibition of inflammation at the tick feeding site from the very beginning of the blood meal. Conversely, sequences related to cement (64P), which function has been correlated with tick attachment, was largely expressed in the mid library.

**Conclusions:**

Our survey provided an insight into the *R. sanguineus *sialotranscriptome, which can assist the discovery of new targets for anti-tick vaccines, as well as help to identify pharmacologically active proteins.

## Background

The brown dog tick, *Rhipicephalus sanguineus*, is a cosmopolitan species from the Ixodidae family [1] found on all continents [2]. Although dogs are the most common host for this tick, it has also been found on other animals, such as cats, rabbits, camels, bovines, goats, horses, sheep, bats, reptiles, and ground feeding birds [1], as well as from humans [3]. It transmits two of the most important arthropod borne pathogens of dogs, namely, *Ehrlichia canis *and *Babesia canis *[4,5].

The saliva of *R. sanguineus *mediates parasitism through components that modulate the innate and acquired immune response of the host [6,7]. Accordingly, these compounds are of major importance for tick survival, helping it feed and evade host defenses, including hemostatic factors and the inflammatory responses [8]. In order to identify protein families relevant for the tick-host interface, salivary transcriptomes (sialotranscriptomes) and microarray analysis of several Ixodid tick species have been done [9-18]. In addition, this strategy can help the identification of proteins from tick saliva that can induce anti-tick resistance and impair or block transmission of tick-borne pathogens [19-22].

Female adult ticks go through a notable succession of changes during feeding and mating. Their body sizes and weights increase gradually as the blood-feeding progresses. During the feeding period their salivary glands undergo a set of qualitative and quantitative alterations in the content of mRNA and protein [17,18]. For *R. sanguineus *female ticks, at days 1-3 (i.e., the early phase of feeding) the change of weight and size is very small, while by the 5th day these parameters are 2 to 3 times greater. After this stage the rapid phase of engorgement (also called late phase) is initiated; the salivary glands start to degenerate and the ticks can reach 50 to100 times the size they were when unfed. The time taken by females of *R. sanguineus *to complete their engorgement is 7-10 days [3].

In the present work, we analyzed the sialotranscriptome of two *R. sanguineus *cDNA libraries, that included transcripts from salivary glands from female ticks fed for 3-5 or 5 days on dogs. A total of 2,034 high quality expressed sequence tags (ESTs) were analyzed producing 1,024 contigs, of which 910 were derived from only one EST. For functional annotation of the unique transcripts we used the BLASTx, comparing them against nine different databases. The comparison of the abundance of ESTs from each contig of the two libraries allowed identification of some genes that were significantly differentially represented. To our knowledge, this work is the first transcriptome analysis of salivary glands of *R. sanguineus *tick species. Moreover, the characterization of components from tick saliva is likely to be of value in future when designing novel methods for the control of ticks and tick-borne diseases, as well as searching for proteins that may have potential use in medical and veterinary pathologies".

## Methods

### Ticks and salivary gland collection

Ticks were obtained from two laboratory colonies, one from the Ribeirão Preto School of Medicine, University of São Paulo, Ribeirão Preto, SP (FMRP-USP), and the other from the School of Agronomical and Veterinary Sciences, São Paulo State University, Jaboticabal, SP (FCAV-UNESP), both were maintained at 29°C in a biochemical oxygen-demand incubator with 85% relative humidity. Adult ticks (25 females and 25 males) were allowed to feed in plastic feeding chambers glued (Britania Adhesives P4104 Latex, Brentwood, UK) to the back of 1-3 years old female mongrel dogs for either libraries. These dogs were not naïve to ticks, however had no ticks when were tick-infested. Tick infestations were performed at both locations (FCAV-UNESP and FMRP-USP) using four dogs (2 per group). After five days, 25 female ticks were collected and used to construct the RsSGL2 library (FCAV-UNESP), while 10 female ticks fed for 3, 4 and 5 days (summing 30 ticks) were pooled and used to make up the RsSGL1 library (FMRP-USP). Salivary glands (25-30 pairs) were dissected from female ticks and washed in ice-cold phosphate-buffered saline (PBS), pH 7.4 and then incubated in RNAlater solution (Ambion, Austin, USA) for 24 h at 4°C and then stored at -80°C until used.

### cDNA library construction and sequencing

Total mRNA was isolated from *R. sanguineus *salivary glands using the micro Fast Track™ 2.0 RNA extraction kit (Invitrogen, Carlsbad, USA) according to the manufacturer's protocol. A long distance PCR based cDNA library was constructed in a λ TripleEx2 vector following the procedures from the SMART™ cDNA Library Construction Kit (Clontech, Palo Alto, USA). This system utilizes oligoribonucleotide (SMART IV) to attach an identical sequence at the 5' end of each reverse-transcribed cDNA strand. The sequence was then employed in subsequent PCR reactions and then digested with restriction enzymes. First-strand synthesis was carried out using PowerScript reverse transcriptase at 42°C for 1 h in the presence of the SMART IV and CDS III (3') primers. Second-strand synthesis was performed by a long-distance (LD) PCR-based protocol using Advantage™ Taq Polymerase (Clontech) mix in the presence of the 5' PCR primer and the CDS III (3') primer. The cDNA synthesis procedure resulted in the creation of *Sfi*I A and B restriction enzyme sites at the ends of the PCR products that are used for cloning into the phage vector.

A small portion of the cDNA obtained by PCR was analyzed on a 1.1% agarose gel with ethidium bromide (1.5 μg/mL). The optimal number of cycles with visible and equally represented products was used. Double-stranded cDNA was immediately treated with proteinase K at 45°C for 20 min, and the enzyme was removed by ultrafiltration though a Microcon (Amicon Inc., Beverly, USA) YM-100 centrifugal filter device. The cleaned, double-stranded cDNA was then digested with *Sfi*I at 50°C for 2 h, followed by size fractionation on a ChromaSpin-1000 column (Clontech).

The cDNA mixture was ligated into the λ TriplEx2 vector (Clontech) and the resulting ligation mixture was packaged using the GigaPack^® ^III Plus packaging extract (Stratagene, La Jolla, CA) according to the manufacturer's instructions. The packaged library was plated by infecting log-phase XL1-Blue *Escherichia coli *cells (Clontech). The percentage of recombinant clones was determined by performing a blue-white selection screening on LB/MgSO4 plates containing X-gal/IPTG. Recombinants were also determined by PCR, using vector primers from the SMART™ cDNA Library Construction Kit (Clontech) and visualizing the products on a 1.1% agarose gel with ethidium bromide. Random clones were sequenced from the 5' direction only, because successful sequencing from the 3' end was usually lower than 40%. Full length sequences were obtained in selected cases by performing primer-based extension protocols. For details see Francischetti et al. and Valenzuela et al. [23,24].

### Bioinformatic tools and statistical tests used

ESTs were trimmed of primer and vector sequences. The BLASTn [25], CAP3 assembler [26] and ClustalW software [27] were used to compare, assemble, and align high quality ESTs, respectively. For functional annotation of the transcripts we used BLASTx [25] to compare the nucleotide sequences with the non-redundant (NR) protein database of the National Center of Biological Information (NCBI) and to the Gene Ontology (GO) database [28]. The program reverse position-specific BLAST (RPS-BLAST) [25] was used to search for conserved protein domains in the Pfam [29], SMART [30], Kog [31], and conserved domains databases (CDD) [32]. We have also compared the transcripts with a subset of mitochondrial/plastid and rRNA nucleotide sequences downloaded from NCBI and to several organism proteomes downloaded from NCBI, ENSEMBL, or VectorBase. For all comparisons please consult Additional file 1. Segments of the three-frame translations of the EST (as the libraries were unidirectional, six-frame translations were not used) starting with a methionine found in the first 30 predicted amino acids, or the predicted protein translation in the case of complete coding sequences, were submitted to the SignalP server [33] to help identify translation products that could be secreted. O-glycosylation sites on the proteins were predicted with the program NetOGlyc [34].

All sequences reported in this paper are available publicly under the accession numbers GT030184-GT032391 and EZ406035-EZ406256 (EST's from adult female salivary gland cDNA library) at GenBank and are accessible in Additional file 1.

For sequence comparisons and phylogenetic analysis, we retrieved tick sequences from GenBank, as well as deduced protein sequences from ESTs deposited in dbEST, as described and made accessible in a previous review article [8]. Phylogenetic analysis and statistical neighbour-joining (NJ) bootstrap tests of the phylogenies were done using the Mega package [35] after sequence alignment was performed using ClustalX [36].

The individual cDNA libraries were directly compared with each other using a customized program (Count Libraries) that assesses the individual contribution of each library to the combined contig. This analysis is interesting to suggest putative proteins that may be over- or under-represented at a given time point. A Chi-square analysis was used to evaluate the significance level at *p *< 0.05 between the number of transcripts in the same contig originating from the two libraries used.

## Results and Discussion

### Overview of the assembled salivary EST set

A total of 2,034 ESTs were used to produce a *R. sanguineus *salivary gland specific transcriptome database (Additional file 1), 875 ESTs from 5 days fed ticks (RsSGL2) and 1,159 from 3 to 5 days fed ticks (RsSGL1), which were assembled yielding 1,024 unique transcripts ("clusters" of related sequences), 910 of which were derived from only one EST (singleton). This large number of singletons contrasts with previous sialotranscriptomes of hematophagous insects and Ixodid ticks, giving an appearance of a "normalized" library.

Manual annotation of the transcripts resulted in seven broad categories of expressed genes (Table 1). The putative housekeeping genes category contained 56% of the clusters, which had, on average, 2.49 sequences per cluster, the secreted category contained 26.6% of the clusters which contained 2.47 ESTs/clusters, while 15.3% of the transcripts, mostly singletons, were not classifiable, constituting the unknown category. The transcripts assigned to the unknown category could represent novel proteins or derive from the less conserved 3' or 5' untranslated regions of genes, as was suggested from the sialotranscriptome of *Anopheles gambiae *[37]. Sequences deriving from *Babesia*, *Anaplasma*, Densovirus and transposable elements (TE) accounted for the remaining sequences, mostly singletons. Babesia and densoviral-related proteins were also described in a previous sialotranscriptome of *Ixodes scapularis *[13]. Ribosomal proteins, possibly derived from *Babesia canis*, were found in the RsGLS1 library, and may be useful for diagnostic purposes (Additional file 1). TE related sequences may either indicate the presence of active transposition in the tick, or more likely, the expression of sequences that are able to suppress transposition [38]. Low level expression of TE sequences have been a relatively common finding in previously analyzed sialotranscriptomes.

**Table 1:** Classification and abundance of salivary transcripts.

Class	ESTs	%	Clusters	ESTs/Cluster
Housekeeping	1138	55.95	457	2.49
Putative secreted	541	26.60	219	2.47
Unknown	312	15.30	306	1.02
Babesia	35	1.72	34	1.03
Transposable elements	5	0.25	5	1.00
Densoviral protein	2	0.10	2	1.00
Anaplasma	1	0.05	1	1.00

**Total**	2034	100	1024	

#### Housekeeping genes

Ninety-eight transcripts (mostly full-length) comprised of 1,138 ESTs were annotated as housekeeping genes and were further categorized into 20 subgroups according to function (Table 2 and Additional file 1). Transcripts associated with protein synthesis machinery represented 47% of all transcripts associated with housekeeping function, an expected result due to the secretory nature of the organ. Energy metabolism accounted for 23% of the transcripts. Eight percent of the transcripts were classified as either 'Hypothetical conserved' or 'Conserved secreted' proteins. These represent highly conserved proteins of unknown function, presumably associated with cellular function yet uncharacterized. This functional distribution is typical and was previously described in other sialotranscriptomes [15,16,39].

**Table 2:** Classification of transcripts associated with housekeeping function.

Class	ESTs	%	Clusters	ESTs/Cluster
Protein synthesis machinery	533	46.84	94	5.67
Metabolism, energy	262	23.02	94	2.79
Unknown conserved	92	8.08	66	1.39
Protein modification machinery	64	5.62	52	1.23
Signal transduction	32	2.81	25	1.28
Protein export	28	2.46	24	1.17
Cytoskeletal	21	1.85	13	1.62
Transcription machinery	20	1.76	16	1.25
Nuclear regulation	18	1.58	15	1.20
Proteasome machinery	15	1.32	11	1.36
Metabolism, carbohydrate	12	1.05	10	1.20
Metabolism, amino acid	7	0.62	7	1.00
Transporters/Storage	7	0.62	4	1.75
Metabolism, lipid	6	0.53	6	1.00
Metabolism, free radicals	6	0.53	5	1.20
Metabolism, dexoxification	4	0.35	4	1.00
Metabolism, nucleotide	4	0.35	4	1.00
Metabolism, intermediate	3	0.26	3	1.00
Extracellular matrix, adhesion	2	0.18	2	1.00
Transcription factor	2	0.18	2	1.00

**Total**	1138	100	457	

#### Putatively secreted class of expressed genes

A total of 541 ESTs, assembled into 219 contigs, were associated with putative *R. sanguineus *salivary secreted components (Table 3 and Additional file 1). These include previously known gene families, such as metalloproteases, lipocalins, protease inhibitor domain-containing peptides, immuno-modulators, antimicrobial peptides, basic-tail, and glycine rich peptides. Several other deduced sequences code for putatively secreted proteins but have poor or non-significant sequence similarity to other known proteins, or to proteins not previously described in tick sialotranscriptomes [8].

**Table 3:** Classification of transcripts that are associated with a secretory function.

Class	ESTs		%	Clusters	ESTs/Cluster
Other putative secreted	192		35.49	103	1.86
Lipocalins	116		21.44	28	4.14
Glycine rich polypeptides	72		13.31	23	3.13
Protease inhibitor domains	56		10.35	29	1.93
Antimicrobial peptides	56		10.35	5	11.20
Immunosuppressors (Evasin/Da-p36)	25		4.62	12	2.08
Metalloproteases	7		1.29	7	1.00
Aegyptin-like	6		1.11	1	6.00
Mucins	3		0.55	3	1.00
Similar to mammal skin proteins	2		0.37	2	1.00
Possible cuticle or cement	2		0.37	2	1.00
Similar to *Grillus *accessory gland peptide	2		0.37	2	1.00
Basic tail	2		0.37	2	1.00

**Total**	541		100	219	

### Detailed analysis of the sialome of *R. sanguineus*

From the sequenced clones, 114 of which code for putative secreted products were meticulously analyzed (Additional file 2). The following presentation is a guide for browsing Additional file 2.

#### Putative secreted proteins with presumed or experimentally validated function

##### Lipocalins

**Figure 1:**
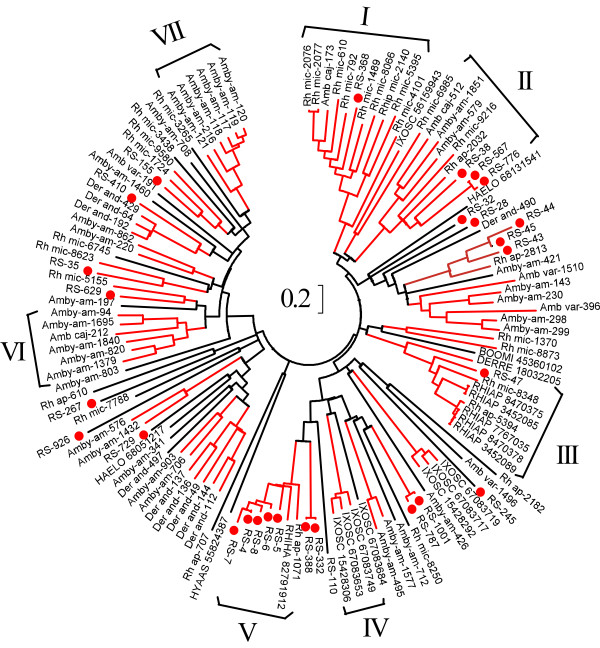
**Relationship of *Rhipicephalus sanguineus *lipocalins to other related tick proteins**. The circular phylogram is based on the alignment of sequences derived from this study and similar sequences obtained from the NR database from NCBI, and from tick sequences derived from dbEST. The red branches have bootstrap support above 75% (10,000 replicates). The bar at the center indicates 20% amino acid divergence. The *R. sanguineus *sequences are indicated by a circle, and start with RS-. The sequences obtained from the NR database are indicated by 5-6 letters related to the tick species followed by the NCBI accession number. Remaining sequences were deduced from dbEST and are available from Francischetti et al. [8].

The lipocalin family of proteins is ubiquitous in animals [40]. Its barrel structure makes it suitable to carry small substances within the cavity and the barrel sides can acquire diverse functions [41]. As an example of convergent evolution, this family has been recruited to serve diverse functions in saliva of ticks and triatomine bugs, where they serve as scavengers of agonists of inflammation and hemostasis, such as ADP [42], biogenic amines [43-45], leukotrienes and thromboxane A_2 _(TXA_2_) [44,46], to carry heme and nitric oxide (NO) [47], anticomplement [48], or anticlotting agents [49]. Typically, dozens of such gene products are found within sialotranscriptomes of ticks and triatomine bugs [8,13,15,16,39,50-52]. Additional file 2 displays 27 lipocalin sequences deduced from the *R. sanguineus *sialotranscriptome, 11 of which are full length. An additional *R. sanguineus *protein sequence similar to tick salivary proteins of ~180 amino acids is annotated as a possible lipocalin. A circular phylogram of the *R. sanguineus *lipocalin sequences containing more than 100 amino acid obtained in this study (26 sequences) as well as homologous sequences obtained from a recently published tick salivary gland database [8] is shown in Figure 1. This phylogram depicts that most of the *R. sanguineus *sequences are dispersed into different clades, which contain sequences from other tick species, suggesting an ancient origin for these genes. Despite this clade dispersion, the phylogram also highlights species specific expansions, as indicated in the clades marked I (*Rhipicephalus (Boophilus) microplus *expansion, including one *R. sanguineus *sequence and one *A. cajennense*), III (*R. appendiculatus *expansion including one *R. sanguineus *and one *R. microplus *sequence), IV (*I. scapularis *expansion), V (*R. sanguineus *expansion including one *R. appendiculatus *and one *R. haemaphysaloides *sequence), VI and VII (both with *Amblyomma americanum *expansions). Other similar expansions can be found by close inspection of Figure 1. These are probably the result of recent gene duplication events [53]. Very few proteins displayed in Figure 1 have been functionally characterized. One of them (RHIAP 8470378) was included in a group of *R. appendiculatus *proteins from clade III, that has been shown to be a scavenger of histamine [54,55]. Interestingly, a *R. sanguineus *sequence named RS-47 is also present in clade III, which suggests it may be a close relative. Rooting with clade III, but lacking significant bootstrap support, the sequence DERRE 18032205, from *Dermacentor reticulatus*, was shown to be a dual binder of histamine and serotonin [43]. An additional possible function for the non-characterized lipocalins found in Figure 1, similar to soft ticks and triatomine, may be to bind adenosine nucleotides, TXA_2, _or leukotrienes.

##### Protease inhibitors

The analysis of the *R. sanguineus *sialotranscriptome revealed several protein sequences containing domains associated with protease inhibitors, such as Kunitz [56], thyropin [57,58] and cystatins [59], as well as unique tick protease inhibitor domains, such as a tick carboxypeptidase inhibitor [60], and a tick anti-thrombin of the madanin/hirudin like family [61].

**Figure 2:**
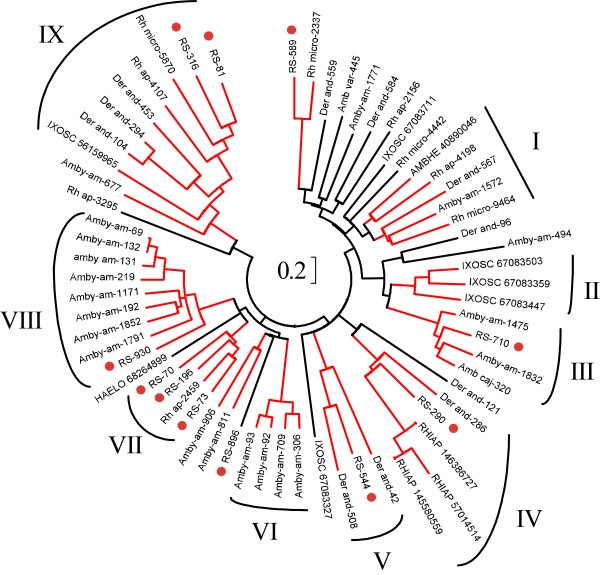
**Relationship of *Rhipicephalus sanguineus *Kunitz domain proteins to other related tick proteins**. The circular phylogram is based on the alignment of sequences derived from this study and homologous sequences obtained from the NR database from NCBI, and from tick sequences derived from dbEST. The red branches have bootstrap support above 75% (10,000 replicates). The bar at the centre indicates 20% amino acid divergence. The *R. sanguineus *sequences are indicated by a circle, and start with RS-. The sequences obtained from the NR database are indicated by 5 letters (3 from the genus and 2 from the species name) followed by the NCBI accession number. Remaining sequences were deduced from dbEST and are available from Francischetti et al. [8].

Kunitz-domain containing proteins, like the lipocalins, are abundantly found in tick sialotranscriptomes. Eleven deduced protein sequences are shown in Additional file 2 which contain one or two Kunitz domains. The circular phylogram of these sequences resulting from the alignment with other related tick sequences (Figure 2) shows that, similarly to the lipocalin family, *R. sanguineus *has several genes coding for the Kunitz family that congregates, with strong bootstrap support, within multi-specific clades (numbered III, IV, V, VII, VIII and IX in Figure 2). Specific gene expansions are also evident (II for *I. scapularis*, VI and VIII for *A. americanum*; and VII for *R. sanguineus*). Clade I, that lacks any *R. sanguineus *sequence, contains the protein from *A. haebraeum *coded by a gene (gi|40890046) that has been previously characterized as a thrombin inhibitor [62] (Clade IV, containing the *R. sanguineus *protein named RS-290, also includes a *R. appendiculatus *sequence (gi|57014514) which has been characterized as a tryptase inhibitor [63]. No other protein shown in Figure 2 has been functionally characterized, although additional Kunitz-domain containing proteins from ticks have been identified as inhibitors of blood clotting [64-69] and platelet aggregation, thus possibly can have a role in the tick feeding process.

Cystatins are cysteine proteinase inhibitors [59] and have been described in the sialotranscriptome of *I. scapularis*, two members of which have been characterized as inhibitors of cathepsins L and S, which play roles in inflammation and immunity [70-72]. These proteins also have been regularly found in sialotranscriptomes of other hard and soft tick species [8]. The *R. sanguineus *sialotranscriptome contained 3 members of this protein family (Additional file 1). Their role as a cysteine proteinase inhibitor remains to be determined.

Thyropin is a domain found as a repeat in the amino terminal region of human thyroglobulin that is proposed to be an inhibitor of cysteine proteases and binding partners of heparin [73,74]. Proteins containing these domains have been reported from other tick sialotranscriptomes [8]. RS-899 is a *R. sanguineus *protein containing 2 thyropin domains, as indicated by its comparison to the Pfam database. No tick thyropins have been functionally characterized to date.

A carboxypeptidase inhibitor, a protein that is rich in cysteins, has been previously reported in *R. bursa*, and postulated to affect fibrinolysis [60,75]. Analysis of our data showed a protein RS-334 that presented match with a carboxypeptidase inhibitor (Additional file 1).

Thrombin inhibitors named madanins were isolated from the tick *Haemaphysalis longicornis *[61]. A related protein named chimadanin is also a thrombin inhibitor [76]. They have no similarities to other proteins found in the NR database. The *R. sanguineus *sialotranscriptome revealed 4 proteins of this family, one of which has a weak similarity to chimadanin, the others being similar to uncharacterized *Amblyomma variegatum *proteins annotated as hirudin-like [77], purported to be a thrombin inhibitor, shown by the ability to inhibit human platelet aggregation stimulated by thrombin. Members of this family were also found in deduced proteins of previously published sialotranscriptomes from metastriate, but not prostriate, ticks [8].

The Kazal motif characterizes many serine protease inhibitors that affects several target proteins, such as thrombin and trypsin [78]. Three related putative peptide sequences from the *R. sanguineus *sialotranscriptome (RS-132, RS-359 and RS-827) matched proteins annotated as Kazal-domain, despite the fact that the *R. sanguineus *proteins themselves lack Kazal domain signature, as searched by rpsBLAST against the conserved domains database.

##### The basic tail and 18.3 kDa superfamily

**Figure 3:**
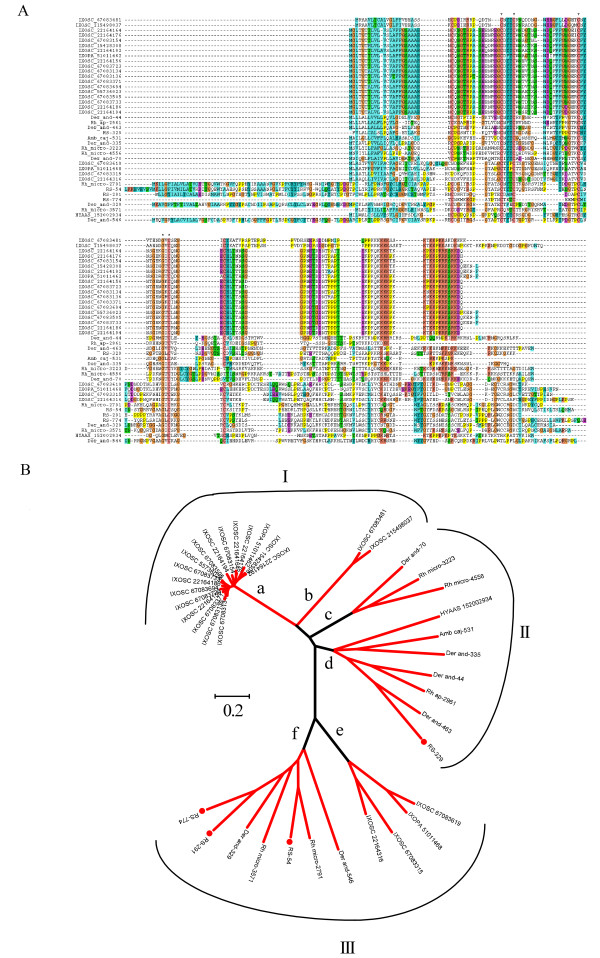
**The salivary basic tail and 18.3 kDa proteins of *Rhipicephalus sanguineus***. A) Clustal alignment with other tick proteins. The asterisk (*) indicates identical amino acids. B) Phylogram of the alignment. The branches shown in red have bootstrap support above 75%. The bar at the centre indicates 20% amino acid divergence. The *R. sanguineus *sequences are indicated by a circle, and start with RS-. The sequences obtained from the NR database are indicated by 5 letters (3 from the genus and 2 from the species name) followed by the NCBI accession number. Remaining sequences were deduced from dbESTand are available from Francischetti et al. [8].

The basic tail family (BT) was so named due to a stretch of lysine residues in the carboxytermini of several related salivary proteins of *I. scapularis *[79]. The cluster of basic amino acids may drive these proteins to negatively charged lipids involved in clotting activation [80,81]. The 18.3 kDa family was found later to be related to the BT family by PsiBLAST [13]. Although more expanded in *Ixodes*, the family is also found in metastriate and argasid ticks [8]. Some proteins of this family in the *I. scapularis *species were characterized as anti-clotting [65]. Four proteins deducted from the *R. sanguineus *sialotranscriptome are divergent but clearly related. They produce matches to basic tail and 18.3 kDa members. Alignment of these proteins and their matches (Figure 3A) reveals that one group of proteins has the typical basic tail signature, including RS-329, while a second group of longer sequences belongs to the 18.3 kDa family, and includes the three remaining *R. sanguineus *sequences. This alignment shows only conservation of one Gly and four Cys residues. The phylogram (Figure 3B) can be divided into three groups and six branches. Group I contains the typical *Ixodes *BT expanded family, with two distinct branches (a and b in Figure 3B). Group II includes metastriate ticks, all containing a BT signature, with two robust clades, represented by branches c and d (Figure 3B). Group III sequences contain typical 18.3 kDa proteins, in two clades represented by branches e and f, e having only *Ixodes *and f including only metastriate sequences. The phylogram clearly demonstrates the evolutionary pathways of this divergent protein family among metastriate and prostriate ticks.

##### Disintegrins

The disintegrins contain an Arg-Gly-Asp (RGD) or Arg-Thr-Ser (RTS) triad flanked by cysteines. These peptides, originally discovered in snake venom, bind to platelet integrins that normally attach to fibrinogen and promote platelet aggregation [82,83]. The *R. sanguineus *sialotranscriptome reveals two members (RS-325 and RS-609) related to this family. RS-325 codes for a 4.7 kDa peptide that has a typical RGD domain, but no similarity to any other known protein. Acquisition of the RGD motif by proteins of other families has been described in antigen-5 salivary proteins from tabanids [84], and in Kunitz peptides of ticks [67]. In addition to its affect on host platelet aggregation, disintegrins may also act on several other inflammatory/immune cell features [85,86], which could decrease host cell migration to the tick-feeding lesion. The transcriptome presented herein also displayed a lipocalin (RS-926) that contains a typical RTS domain of the disintegrin family [87], which was not found in any other member of the lipocalin family, suggesting a possible additional function. Similarly, the Kunitz containing proteins RS-316 and RS-589 also each have a RTS and a KTS motif surrounded by cysteines.

##### Cys-rich peptides associated with metalloproteases

Metalloproteases often have extra domains that may interact with matrix proteins [88]. Tick sialotranscriptomes revealed Cys rich proteins that are similar to these extra domains of metalloproteases, including the expanded ixostatin family in *I. scapularis *and *I. pacificus *[11,13]. RS-707 codes for a 14.8 kDa mature protein of that is similar to other Cys rich metastriate proteins. Their function has not been characterized.

##### Immunomodullatory and antiinflammatory proteins

Tick saliva has been known to have immunomodulatory activity for decades now [89-91]. More recently, unique proteins have been characterized that act directly on immune cells, or in complexing and annihilating the effect of cytokines [92,93].

Dendritic cells pre-exposed to *R. sanguineus *tick saliva showed reduced migration towards chemokines CCL3 and CCL4 [94]. These results lead to the discovery of the family of Evasin proteins, which are chemokine binding molecules isolated from *R. sanguineus *tick saliva [90] that inhibit inflammation and dendritic cell migration [95,96]. Evasin-1 (gi|215275254) binds to chemokines CCL3, CCL4 and CCL18 and corresponds to the contig RS-77 (Additional file 2). Evasin-3 (gi|215275255) binds to chemokines CXCL1 and CXCL8, corresponding to RS-60. Evasin-4 (gi|215275256) binds to chemokines CCL5 and CCL11 and corresponds to RS-909. The *R. sanguineus *sialotranscriptome revealed five additional Evasin sequences (RS-95, RS-119, RS-216, RS-391 and RS-505). These Evasins group into two families, family 1: contains Evasins-1 and -4 and present the conserved block C-x(14,16)-C-x(3)-C-x(9,18)-C-x(15,18)-Y-x-C-x(2)-G-x-C-x-N-x(2,3)-C-x(8)-C, while family 2: contains Evasin-3 and the conserved motif C-x(3)-C-x(2,5)-G-x(3,4)-C-P-x(1,2)-G-x(0,1)-C-x-C.

**Figure 4:**
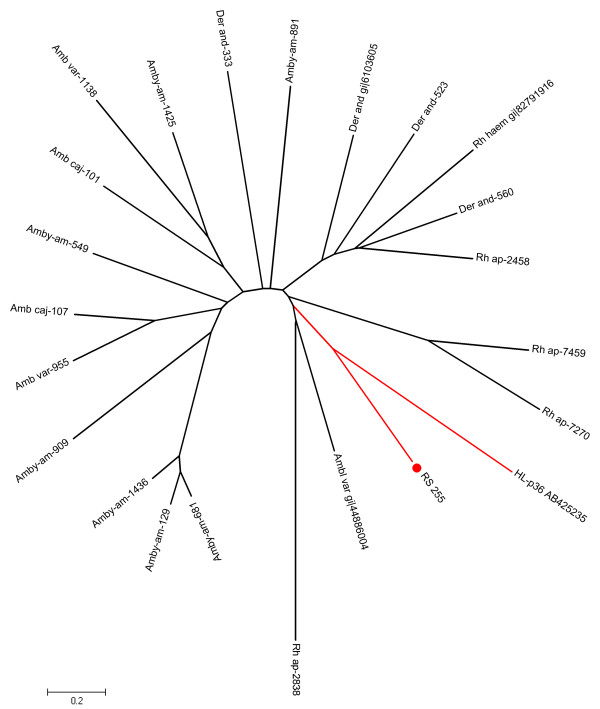
**The immunosuppressive protein related to other tick species**. The *R. sanguineus *sequence is indicated by a circle, and start with RS-. The remaining sequences were derived from the NR database and are indicated by 5 letter followed by the NCBI accession number. The protein sequences were aligned using the Clustal program and the dendrogram was created using the Mega package after 10,000 bootstrap replicates using the neighbour joining (NJ) algorithm. The bar at the bottom represents 20% of amino acid substitution. Remaining sequences were deduced from dbEST and are available from Francischetti et al. [8].

The transcriptome presented herein contained a 3' truncated protein coded by RS-255 that matched tick proteins deposited in the NR database annotated as "similar to Da-p36". Da-p36 was isolated from *Dermacentor andersoni *and the recombinant protein inhibited lymphocyte proliferation [97]. Another immuno-suppressive protein, isolated from *H. longicornis*, HL-p36, also showed an anti-proliferative cell effect that was related to down-regulation of mRNA levels for IL-2 [98]. The phylogram of the *R. sanguineus *sequence resulting from the alignment with other immunosuppressive tick sequences shows that RS-255 is more closely related to the HL-p36 than to sequences of other ixodid ticks (Figure 4).

##### Defensins

**Figure 5:**
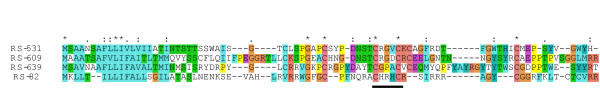
**The salivary defensins of *Rhipicephalus sanguineus *were aligned using ClustalW**. The symbols at the top represent identity (*), conserved (:) and weakly conserved (.) amino acids. The bar below the alignment indicates the region where the Arg-Gly-Asp (RGD) triplet flanked by cysteines is found on RS-609.

Defensins are ubiquitous peptides with antimicrobial properties [99,100]. For ticks, the presence of defensins suggest roles in protection from pathogens. The sialotranscriptome of *R. sanguineus *revealed four divergent members of this family, one of which, as indicated above, contains an RGD motif (Figure 5). RS-82 is similar to other tick defensins, while the remaining are much more divergent; RS-531, for example, was found to be most similar to a wheat defensin.

##### GY (Gly-Tyr) rich peptides

Salivary transcriptomes of haematophagous arthropods, including ticks have revealed the presence of 10 kDa secreted peptides containing multiple GY repeats [51]. Similar peptides in *Caenorhabditis elegans *were shown to have antimicrobial activity [101]. The *R. sanguineus *sialotranscriptome contained three transcripts coding for peptides containing GY repeats, two of which have less than 60 amino acids and are distantly related (RS-11 and RS-76). They present similarities to tick and worm peptides deposited in the NR database, as well as to several peptides deduced from ESTs present in other tick transcriptomes deposited in dbEST. RS-79 codes for a larger peptide homologous to other GY rich proteins of arthropods, including some annotated as egg-shell proteins. The abundance of Tyr residues may provide for cross linking of these peptides upon phenol oxidase activity. In arthropods, these enzymes participate in sclerotizing the proteins in the flexible exoskeleton after a molt [102,103].

##### Glycine-rich/Cement proteins

Ticks attach to their hosts with the help of specialized mouthparts and remain attached by the secretion of cement proteins that glues the mouthparts into the host's skin [104]. Some of these proteins have been characterized and tested as anti-tick vaccines [105-108]. Tick salivary Gly rich proteins are derived from several gene families, some of which are similar to spider fibroin [8]. The *R. sanguineus *sialotranscriptome contained seven full length proteins of this generic family, plus eight fragments (Additional file 1).

##### Mucin/Perithrophin

Mucins are proteins containing galactosylation of Ser or Thr residues, and are normally found associated with mucosal membranes where they may play a role in the immune response [109,110]. Sialotranscriptomes of ticks and other blood feeding arthropods regularly display such proteins, often with a chitin binding domain that might coat the food canals with a mucous lubricant, in addition to functioning in extracellular matrix adhesion [13,49]. RS-676, similar to arthropod proteins annotated as mucins and peritrophins, contains five putative glycosylation sites near the carboxy terminus and a chitin binding domain (Additional file 2). RS-843 and RS-588 are related proteins with 11 putative glycosylation sites each. These proteins only provide poor matches to other proteins when queried using the program BLASTp against the NR database.

#### Putative secreted proteins with uncharacterized function

##### 8.9 kDa family

**Figure 6:**
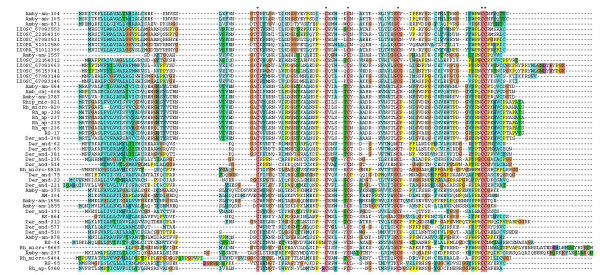
**Alignment of selected members of the 8.9 kDa family of hard ticks**. The asterisk (*) shows the 6 conserved cysteines. The sequences obtained from the NR database are indicated by 5 letters (3 from the genus and 2 from the species name) followed by the NCBI accession number. Remaining sequences were deduced from dbEST and are available from Francischetti et al. [8].

Sixty members of this protein family have been identified from prostriate and metastriate ticks as described in a recent review [8]. The *R. sanguineus *sialotranscriptome reveals four additional members of this family, identified by sequence comparison to the NR database. Two of these members, RS-17 and RS-864 are closely related to each other. Alignment of selected members of this family shows a conserved group of six cysteine residues, including a doublet at the carboxytermini (Figure 6). Some members of the family have an additional two cysteines. PsiBLAST of members of the 8.9 kDa family against the NR database plus the deduced proteins described in [8] identified *Drosophila *proteins of similar sizes which have a similar Cys framework, including a doublet at the carboxyterminus. The BLAST link of a *D. melanogaster *protein (gi|162951779) is interesting, as it shows various *Drosophila *proteins, as well as a *Culex quinquefasciatus *protein that are very similar to a previously described sialoprotein from *Aedes albopictus*, and also a secreted salivary protein from *I. Scapularis*, which is a member of the 8.9 kDa family. The function of this protein family in *Drosophila *is still unknown, as reported by FlyBase [111].

##### 5.3 kDa family

This family of peptides was initially found in *I. scapularis*, where some members were up regulated in ticks infected with *Borrelia burgdorferi*, suggesting a role in immune responses to bacteria [13]. Two sequences (RS-968 and RS-402) of the *R. sanguineus *sialotranscriptome matched with this family.

##### Metastriate one-of-each family

**Figure 7:**
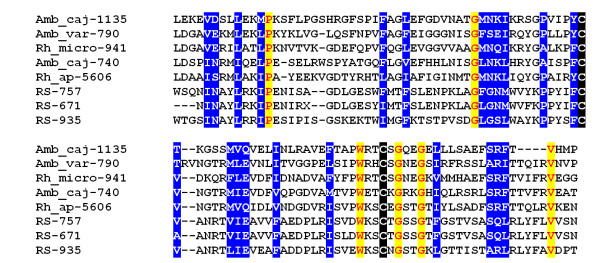
**Alignment of the one-of-each family of metastriate proteins**. Blocks of identical amino acids are shown in yellow background; conserved cysteines are shown in black background; other conserved amino acids are shown in blue background. Sequences from *R. s sanguineus *start with RS-. Other sequences were obtained from Francischetti et al. [8].

While many tick salivary proteins belong to multi gene families, a previous family, specific to metastriates, was described which appears to have only one member per metastriate species. The *R. sanguineus *sialotranscriptome seems to break this rule by providing evidence for three proteins of this family (RS-757, RS-671 and 935). Alignment of these three proteins with other similar metastriate proteins reveals absolute conservation of two cysteines, one tryptophan, one proline, three glycines and one valine residues, plus several other conserved substitutions (Figure 7). PsiBLAST of the *R. sanguineus *sequences against the NR database with the addition of the deduced tick proteins described before [8] retrieves only hard tick protein, suggesting that this protein family definitely belongs to Ixodidae.

##### Metastriate acid tail family

RS-907 and RS-881 are similar to *R. microplus *and *Amblyomma *proteins that have an acidic tail. PsiBLAST of these proteins against the NR plus tick protein data sets recovers only tick proteins thus this appears to be a tick specific protein with unknown function.

##### Other putative secreted proteins

Additional file 2 describes 11 proteins annotated as putative secreted. Some of them match previously described tick proteins that have not been characterized as a protein family due to lack of members in different species. It is possible that they may be recognizable as members of protein families as more transcriptomes/genomes are annotated, or they may represent *R. sanguineus *proteins resulting from genes under accelerated divergent evolution. It should also be noted that some of these proteins may represent annotation artifacts of 3' UTR's, or may represent the truncated carboxyterminus of known proteins, because their membrane domains will often appear as a signal peptide. Additionally, four proteins with putative signal peptide were highly conserved, and accordingly, may represent housekeeping proteins with hormonal or extracellular matrix functions.

### Differential expression among the two libraries

The EST abundance and assembly derived from two libraries, one made of mRNA from ticks feeding for 3-5 days (early to mid library, RsSGL1) and the other from ticks feeding for 5 days (mid library, RsSGL2) is depicted in Additional file 1. Comparison of the abundance of ESTs contributing to each contig in Count Libraries by chi square analysis allowed for the identification of some genes that are significantly differentially represented among the two libraries.

Among the putative lipocalins, three presented an alternating pattern of expression (Table 4): RS-4 and RS-6, both corresponding to gi82791912, were over expressed in the mid library (with 17 and 12 ESTs in the mid library and only 8 and 1 ESTs in the early to mid library); other putative lipocalins RS-32, corresponding to gi68131541, was over expressed in the early to mid library (no ESTs versus 9). This suggests that the tick possibly relies on an escape mechanism for ligands of its lipocalins throughout the female's blood meal by means of antigenically distinct, but functionally similar proteins. A putative thrombin inhibitor RS-20, was over expressed in the early to mid library (with 16 ESTs derived from the early to mid library and only one from the mid library). This pattern is compatible with a need to suppress coagulation during the initial stages of the blood meal. Unexpectedly, a glycine rich protein RS-23 (64P), similar to cement, was over expressed in the mid library (13 versus 1 EST). This time of expression for a cement protein is peculiar for our libraries, since other work shows down regulation already within four days of tick feeding [112]. A possible explanation can be that this protein has an additional function in this phase, yet this requires more investigation. Regarding the Evasins, differences in expression did not reach significance for any member of this class of immunomodulators. However, as a class, they were expressed more abundantly in the early to mid library (20 versus 5 ESTs). This finding underscores the tick's need to avoid the cellular inflammatory responses triggered by its insults to the skin at the very beginning of the blood meal. This finding is also compatible with the function of Evasins as chemokine-binding proteins. Two genes (RS-17 and RS-40) encoding proteins of unknown function were more abundantly expressed in the early to mid library. There were also mitochondrial products possibly coding for rRNA that were differentially expressed: RS-18 was over expressed early to mid (22 versus 1 EST), while RS-2 was over expressed later (23 versus 0 EST) (Additional file 1). This temporal difference in gene expression was previously described in *I. scapularis *[13].

**Table 4:** Differentially expressed transcripts between the RsSGL1 and RsSGL2 cDNA libraries.

Number of Contig	Best match of Over expressed Cluster to NR protein database Probable secreted class of protein	*E *value	No of ESTs RsSGL1	No of ESTs RsSGL2	Expected	***p *value **χ^**2 **^**test**
	**Protease inhibitor domain-containing**					
Contig-20	gi67968373 chimadanin Thrombin inhibitor [*Haemaphysalis longicornis*]	3e-006	**16**	**1**	12.85	*0.00094*

	**Lipocalins**					
Contig-4	gi82791912 putative serotonin and histamine binding protein [*Rhipicephalus haemaphysaloides haemaphysaloides*]	8e-068	**8**	**17**	18.90	*0.00067*
Contig-6	gi82791912 putative serotonin and histamine binding protein [*Rhipicephalus haemaphysaloides haemaphysaloides*]	3e-067	**1**	**12**	9.83	*0.00087*
Contig-32	gi68131541 hypothetical protein [*Haemaphysalis longicornis*]	5e-006	**9**	**0**	6.80	*0.00908*

	**Glycine rich proteins/other cement related sequences**					
Contig-23	gi20069012salivary gland-associated protein 64P [*Rhipicephalus appendiculatus*]	2e-022	**1**	**13**	10.58	*0.00051*

	**Immunomodullatory-antiinflammatory proteins**					
Contig-60	gi215275255EVA3_RHISA RecName: Full = Evasin-3	1e-033	**6**	**0**	4.53	*0.03316*

	**Other putative salivary peptides**					
Contig-17	gi215497897 secreted protein, putative [*Ixodes scapularis*]	8e-005	**24**	**5**	21.93	*0.00030*
Contig-40	gi76786687 putative secreted protein [*R. microplus*]	0.002	**8**	**0**	6.05	*0.01391*

RsSGL1 - salivary gland cDNA library made from female ticks fed for 3-5 days (early to mid library).

RsSGL2 - salivary gland cDNA library made from female ticks fed for 5 days (mid library).

## Conclusions

Analysis of the sialotranscriptome of two *R. sanguineus *cDNA libraries, from RsSGL1 and RsSGL2, identified many transcripts coding for different components that can favor the tick in detriment of the host. Some were common to both libraries, such as protein sequences associated with proteases inhibitors, disintegrins with RGD, RST and KTS motifs, immunomodullatory and antiinflammatory proteins, such as Evasins and Da-p36, as well as basic tail and 18.3 kDa proteins, mucins, defensins and antimicrobial peptides. An additional phylogenetic analysis indicated conservation between protein families, a phenomenon also found in other tick species, in particular expansion of the lipocalin and Kunitz superfamilies. The phylograms also indicated species specific expansions that probably result from recent gene duplication events, suggested as of important evolutionary adaptive value [13,113]. Moreover, the phylogenetic trees depict that most of the *R. sanguineus *sequences are dispersed into different clades, which contain sequences from other tick species, suggesting an ancient origin for these genes. One of the phylogram also clearly demonstrates the evolutionary pathways of 18.3 kDa protein family are divergent among metastriate and prostriate ticks. Furthermore, we found that the transcript RS-255 codes for a sequence closely related to a recently identified transcript found in *H. longicornis *that codes for a protein that is similar to the immunosuppressant protein Da-p36.

Of interest, we observed that many genes were significantly differentially represented among the early to mid library (RsSGL1) and mid library (RsSGL2). Two transcripts related with lipocalin were over expressed, whereas one was down expressed in the mid library. Thrombin inhibitor and Evasins were over expressed in the early to mid library, while unexpectedly sequences related to cement (64P) were mostly expressed in the mid library. These differences possibly represent adaptations of the tick to the dynamics of the host's anti-homeostatic responses to tick feeding. However, mentioned differences require more detailed examination.

Taken together, these results improve our knowledge of the salivary components of the *R. sanguineus *that can lead to a better understanding of parasite-host interactions, and may originate innovative strategies to find candidate antigens for vaccines, as well as help to discover drugs that could give support to treat coagulopathies and, inflammatory and immunological disorders.

Note: All sequences reported in this paper are available publicly under the accession numbers GT030184-GT032391 and EZ406035-EZ406256 (EST's from adult female salivary gland cDNA libraries) at GenBank.

## Authors' contributions

EA, BRF, LGB and SRM constructed and sequenced the libraries; JGV assisted with the strategy of library construction; BRF devised the experimental design for collection of samples; EA and JMA performed the bioinformatic treatment of the sequences; JMR, EA, BRF and IKFMS performed the analyses, including phylogenetic and statistical analyses, and drafted the manuscript; BRF, JSS and IKFMS participated in the study's coordination. All authors read and approved the final manuscript.

## Supplementary Material

Additional file 1 **Hyperlinked Microsoft Excel file with assembled EST's and various database comparisons**. The EST assembly, BLAST, and signal peptide results were loaded into an Excel spreadsheet for manual annotation. Click here for file

Additional file 2 **Hyperlinked Microsoft Excel file with coding sequences and various database comparisons**. 114 of which code for putative secreted products were meticulously analyzed in the library of female salivary glands of *R. sanguineus*. Click here for file
